# Guideline-directed medical therapy for HFrEF: sequencing strategies and barriers for life-saving drug therapy

**DOI:** 10.1007/s10741-023-10325-2

**Published:** 2023-06-14

**Authors:** Jishnu Malgie, Pascal R. D. Clephas, Hans-Peter Brunner-La Rocca, Rudolf A. de Boer, Jasper J. Brugts

**Affiliations:** 1 Department of Cardiology, Erasmus MC University Medical Center, Rotterdam, The Netherlands; 2 Department of Cardiology, Maastricht University Medical Centre, Maastricht, The Netherlands

**Keywords:** Registry, Pharmacotherapy, Implementation, Guidelines, Sequencing, Titration

## Abstract

Multiple landmark trials have helped to advance the treatment of heart failure with reduced ejection fraction (HFrEF) significantly over the past decade. These trials have led to the introduction of four main drug classes into the 2021 ESC guideline, namely angiotensin-receptor neprilysin inhibitors/angiotensin-converting-enzyme inhibitors, beta-blockers, mineralocorticoid receptor antagonists, and sodium-glucose cotransporter-2 inhibitors. The life-saving effect of these therapies has been shown to be additive and becomes apparent within weeks, which is why maximally tolerated or target doses of all drug classes should be strived for as quickly as possible. Recent evidence, such as the STRONG-HF trial, demonstrated that rapid drug implementation and up-titration is superior to the traditional and more gradual step-by-step approach where valuable time is lost to up-titration. Accordingly, multiple rapid drug implementation and sequencing strategies have been proposed to significantly reduce the time needed for the titration process. Such strategies are urgently needed since previous large-scale registries have shown that guideline-directed medical therapy (GDMT) implementation is a challenge. This challenge is reflected by generally low adherence rates, which can be attributed to factors considering the patient, health care system, and local hospital/health care provider. This review of the four medication classes used to treat HFrEF seeks to present a thorough overview of the data supporting current GDMT, discuss the obstacles to GDMT implementation and up-titration, and identify multiple sequencing strategies that could improve GDMT adherence.

**Graphical Abstract:**

Sequencing strategies for GDMT implementation. GDMT: guideline-directed medical therapy; ACEi: angiotensin-converting enzyme inhibitor; ARB: Angiotensin II receptor blocker; ARNi: angiotensin receptor–neprilysin inhibitor; BB: beta-blocker; MRA: mineralocorticoid receptor antagonist; SGLT2i: sodium–glucose co-transporter 2 inhibitor
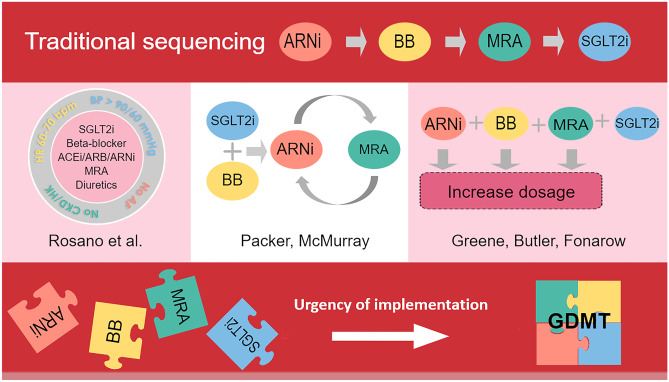

## Introduction

Over the past decades, the treatment of heart failure with reduced ejection fraction (HFrEF) has advanced considerably. Despite remarkable progress regarding both pharmacological and device therapy, heart failure (HF) remains an important healthcare burden with the latest trials still showing a cardiovascular mortality rate of around 10–15% after 2 years of follow-up [1, 2]. Additionally, the current 1–2% prevalence of HF in the Western World is projected to increase in the future [3], highlighting the need for best therapy in individuals with HF. The 2021 European Society of Cardiology (ESC) HF guideline recommends initiating four main drug classes, namely [1] angiotensin receptor-neprilysin inhibitors (ARNi), [2] beta-blockers (BB), [3] mineralocorticoid receptor antagonists (MRA), and [4] sodium-glucose cotransporter-2 inhibitors (SGLT2i) [4]. Although multiple landmark randomized controlled trials have proven the life-saving effect of these therapies [1, 2, 5–17], their timely integration at target dose remains insufficient in real-world clinical practice [18, 19]. Novel sequencing and implementation strategies intend to reduce the delay of drug initiation and strive to further optimize the titration process [20–22]. This review about guideline-directed medical therapy (GDMT) for HFrEF aims to provide a comprehensive overview of the evidence that current GDMT and their target doses are based on, describe the known barriers for GDMT implementation, and identify and summarize sequencing and implementation strategies that could improve GDMT adherence.

## Medical therapy as recommended by the 2021 European Society of Cardiology guideline

### Drug class I: Angiotensin converting enzyme inhibitor/angiotensin-receptor (neprilysin) inhibitor (ACE/ARB/ARNi)

Angiotensin converting enzyme inhibitors (ACEi) were the first drug class with solid evidence of reducing mortality and morbidity in HFrEF, based on trials such as CONSENSUS and SOLVD-Treatment [5, 6]. Therefore, ACEi have been a recommended treatment for HFrEF since 1997 [23]. While the VAL-HeFT and CHARM-added trials initially analyzed the add-on effect of ARB on top of ACEi, the CHARM-alternative trial later found that ARB are a good ACEi alternative in ACEi intolerant patients [8, 9, 17]. In terms of the dose–response relationship of ACEi/ARB, a meta-analysis has shown a slight reduction in mortality favoring higher doses, although no difference in hospitalization rate was observed [24]. In the PARADIGM-HF trial, ARNi was found to reduce all-cause mortality and hospitalization rates, leading to its recommendation over ACEi for HFrEF patients [4, 16]. However, it should be noted that only patients who could tolerate ACEi were eligible for the PARADIGM-HF trial. Also, a run-in period was employed to assess patient tolerability for ARNi, which 12% did not complete. The true tolerability of ARNi in real-world patients is therefore still not fully known. Regarding the dose–response effect of ARNi, a post-hoc analysis of the PROVE-HF trial analyzed patients at target dose versus sub-target, but maximally tolerated, dose [25]. Similar relative decreases in biomarkers and similar amounts of reverse cardiac remodeling were found between the groups, although the study did not report on mortality and hospitalization rates. Therefore, the tolerability and dose–response effect of ARNi in real-world patients still require more research.

### Drug class II: beta-blockers (BB)

Around the change of the century, BB were added on top of ACEi as the second drug class for HFrEF [26–28]. The CIBIS-II, MERIT-HF, and COPERNICUS trials all demonstrated BB to significantly reduce all-cause mortality [12–14]. However, target dose was only reached by 2/3 of patients in the latter two studies, with neither study reporting on reasons for not reaching target dose. Later, the SENIORS trial demonstrated the same benefits in elderly patients (age > 70) [15]. The MOCHA trial reported a dose–response effect of carvedilol in terms of left ventricular ejection fraction (LVEF) improvement, alongside reduced mortality and hospitalization rates [29]. While a significant effect on NYHA class was not found, up-titrating beta-blockers is currently encouraged in order to improve survival rates [4].

### Drug class III: mineralocorticoid receptor antagonists (MRA)

The RALES and EMPHASIS-HF trials resulted in the addition of MRA as the third drug class for HFrEF [10, 11, 30]. Preventing the effects of aldosterone by competitively binding to the mineralocorticoid receptor proved to be an effective addition to ACEi, mostly due to ACEi not sufficiently inhibiting aldosterone production. Initially, the RALES trial found that in NYHA class III/IV patients, spironolactone significantly reduced all-cause mortality and hospitalization rates [10]. The authors reported a dose of 12.5 to 25 mg of spironolactone to be pharmacologically active in conjunction with ACEi and therefore recommended starting at 25 mg daily. Subsequently, the EMPHASIS-HF trial found similar benefits in patients with NYHA class II [11]. Up-titrating to 50 mg was recommended in case of persisting symptoms in patients without hyperkalemia [10]. Although convincing dose–response data for MRA > 25 mg daily is lacking, the 2021 ESC HF guideline nonetheless recommends up-titrating both spironolactone and eplerenone to 50 mg [4, 31].

### Drug class IV: SGLT2 inhibitors

The fourth and currently latest drug class are SGLT2i. The DAPA-HF trial found a reduction in all-cause mortality and a reduction in worsening HF events, favoring the dapagliflozin group [2]. Of note, less than 11% of patients were on ARNi therapy, but ACEi/ARB prescription rates were high. A year later, the EMPEROR-Reduced trial found that empagliflozin significantly reduced the composite endpoint of CV mortality or worsening HF. ARNi prescription rates were slightly higher compared with the DAPA-HF trial, at around 20% for both groups. Importantly, a meta-analysis of both trials has revealed a 13% decrease in all-cause mortality and a 25% reduction in the composite endpoint of recurrent heart failure hospitalizations or cardiovascular death [32]. Furthermore, since the starting dose is equivalent to the target dose used in the randomized controlled trials (RCTs), SGLT2i do not require further dose titration. An overview of the historical ESC HF guideline recommendations is provided in Table 1.

**Table 1:** Evolution of the recommendations for the four main drug classes according to the ESC HF guideline

**ESC guideline year**	**ACEi**	**BB**	**MRA**	**ARB**	**ARNi**	**SGLT2i**
1997 [23]	All symptomatic patients with LV systolic dysfunction, or if asymptomatic with LVEF < 35%	Only for idiopathic dilated cardiomyopathy, or when ischemia is suspected	MRA may be added in severe HF to improve diuresis. Should generally not be used in conjunction with ACEi, only if hypokalemia persists despite ACEi	-	-	-
2001 [26]	Level A: all patients with LVEF < 40–45%	Level A: NYHA II-IV patients on ACEi and diuretics Level B: all patients after MI in addition to ACEi	Level B: NYHA III-IV, in addition to ACEI and diuretics	Level C: Alternative for symptomatic patients intolerant to ACEi Level B: In combination with ACEi for worsening HF	-	-
2005 [27]	1A: all patients with LVEF < 40–45%	1A: NYHA II-IV patients on ACEi and diuretics 1B: Asymptomatic patients after MI	1B: NYHA III-IV, despite BB, ACEi, and diuretics 1B: in addition to ACEi and BB in HF after MI	1B: Alternative for symptomatic patients intolerant to ACEi 1A: can be considered in symptomatic patients as addition to ACEi	-	-
2008 [28]	1A: all HFrEF patients	1A: NYHA II-IV, or asymptomatic after MI	1B: NYHA III-IV and lVEF < 35%, despite BB and ACEi or ARB	1A: symptomatic HFrEF patients despite ACEi and BB 1B: Recommended as alternative for symptomatic patients intolerant to ACEi	-	-
2012 [30]	1A: all HFrEF patients in addition to BB	1A: all HFrEF patients in addition to ACEi	1A: NYHA II-IV and LVEF < 35%, despite ACEi and BB	1A: HFrEF patients intolerant to ACEi 1A: NYHA II-IV HFrEF patients, despite ACEi and BB and intolerant to MRA	-	-
2016 [40]	1A: all HFrEF patients in addition to BB	1A: symptomatic HFrEF patients, in addition to ACEi 1B: asymptomatic LV dysfunction after MI	1A: symptomatic and LVEF < 35%, despite ACEi and BB	1B: symptomatic patients intolerant to ACEi IIB: may be considered if symptomatic, despite BB and intolerant to MRA	1B: recommended as replacement for ACEi in symptomatic patients, despite ACEi, BB, and MRA	2A: empagloflozin should be considered as HF prevention for DM2 patients
2021 [4]	1A: all HFrEF patients	1A: stable HFrEF patients	1A: all HFrEF patients	1B: symptomatic patients intolerant to ACEi/ARNi	1B: as replacement for ACEi in all HFrEF patients	1A: all HFrEF patients

*LV* left ventricle, *LVEF* left ventricular ejection fraction, *HF* heart failure, *HFrEF* heart failure with reduced ejection fraction, *NYHA* New York Heart Association, *ACEi* angiotensin-converting enzyme inhibitor, *ARB* angiotensin II receptor blocker, *ARNi* angiotensin receptor–neprilysin inhibitor, *BB* beta-blocker, *MRA* mineralocorticoid receptor antagonist, *SGLT2i* sodium–glucose co-transporter 2 inhibitor

## Additive effect of all four drug classes

**Fig. 1:**
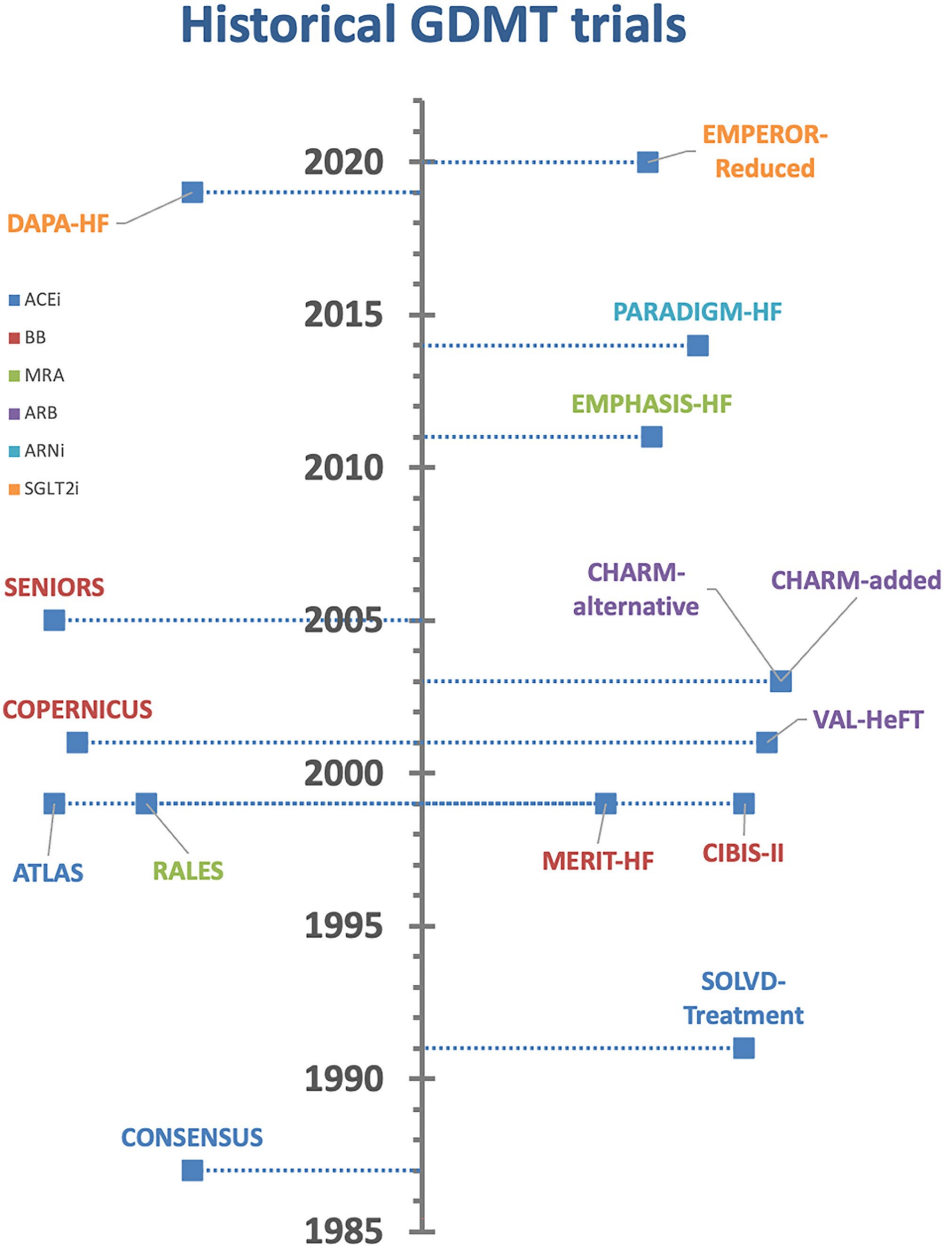
Historical GDMT trials. GDMT, guideline-directed medical therapy; ACEi, angiotensin-converting enzyme inhibitor; ARB, angiotensin II receptor blocker; ARNi, angiotensin receptor–neprilysin inhibitor; BB, beta-blocker; MRA, mineralocorticoid receptor antagonist; SGLT2i, sodium–glucose co-transporter 2 inhibitor

In the aforementioned landmark trials (see Fig. 1), each new drug class has been proven to be effective on top of the previously introduced drug classes. This implies the additive effect of each drug class, which was to be expected considering the different pathways and targets of each drug. However, it is paramount to recognize that this additive effect does not necessarily mean the use of background therapy is required to achieve a treatment effect for the subsequent drug class [33]. For example, the CIBIS III trial showed it is equally effective to start a BB as initial therapy instead of ACEi [34]. Additionally, when the 1999 RALES trial was conducted BB were not yet standard of care, while around 87% of patients were on BB therapy in the EMPHASIS-HF trial [10, 11]. Since endpoint reductions were similar between the trials, this implies that background therapy with a BB is not necessary for achieving a treatment effect with MRA. Likewise, the treatment effects of SGLT2i in both DAPA-HF and EMPEROR-reduced were comparable, whether or not patients were on ARNi background therapy [1, 2, 32]. On the other hand, some treatments that were common practice during several trials have been largely abandoned. For instance, digoxin was background therapy in 30–65% of all patients in the US Carvedilol, CIBIS, and MERIT-HF trials, but the percentage of HF patients using digoxin has since declined to < 10% [13, 14]. Strictly speaking, we have insufficient data to know for sure if beta blockers are efficacious in patients without digoxin. However, no interaction was found in any of the trials with the presence or absence of other treatment. An overview of the evidence for drug class independence is given in Table 2 [20].

**Table 2:** Early effects of foundational treatments on major outcomes in large-scale trials. Reprinted from Eur J heart fail, volume 23, issue 6, packer and McMurray, rapid evidence-based sequencing of foundational drugs for heart failure and a reduced ejection fraction, Pages 882–894, Copyright 2023, with permission from Wiley

**Drug class**	**Trial**	**Distinguishing feature**	**Endpoint reported**	**Hazard ratio (95% CI)**
Beta-blockade following acute myocardial infarction (with or without ACE inhibitors)	BHAT	None receiving ACE inhibitors	All-cause mortality	≈ 0.73
	CAPRICORN (carvedilol)	Most receiving ACE inhibitors		0.77 (0.60–0.98)
ACE inhibitors (with or without beta-blockade)	SAVE (captopril)	Post-infarction patients with LV systolic dysfunction, 35–40% on beta-blockers	All-cause mortality	0.81 (0.68–0.97)
	SOLVD Treatment (enalapril)	Heart failure with LV systolic dysfunction, no use of beta-blockers		0.84 (0.74–0.95)
ACE inhibitors (with or without mineralocorticoid receptor antagonists)	CONSENSUS (enalapril)	> 50% receiving mineralocorticoid receptor antagonist	All-cause mortality	≈ 0.73
	SOLVD Treatment (enalapril)	No recorded use of mineralocorticoid receptor antagonist		0.84 (0.74–0.95)
Mineralocorticoid receptor antagonists (with or without beta-blockade)	RALES(spironolactone)	≈ 10% on a beta-blocker	All-cause mortality	0.70 (0.60–0.82)
	EMPHASIS-HF(eplerenone)	> 85% on a beta-blocker		0.76 (0.62–0.93)
Sacubitril/valsartan (with or without mineralocorticoid receptor antagonists)	PARADIGM-HF	Receiving mineralocorticoid receptor antagonist	Cardiovascular death	0.84 (0.73–0.98)
		Not receiving mineralocorticoid receptor antagonist		0.75 (0.63–0.89)
SGLT2 inhibitors (with or without neprilysin inhibitors)	DAPA-HF and EMPEROR-Reduced	Receiving neprilysin inhibitor	Cardiovascular death or hospitalization for heart failure	0.68 (0.53–0.89)
		Not receiving neprilysin inhibitor		0.75 (0.68–0.84)

*ACE* angiotensin-converting enzyme, *CI* confidence interval, *LV* left ventricular, *SGLT2* sodium-glucose co-transporter 2

**Fig. 2:**
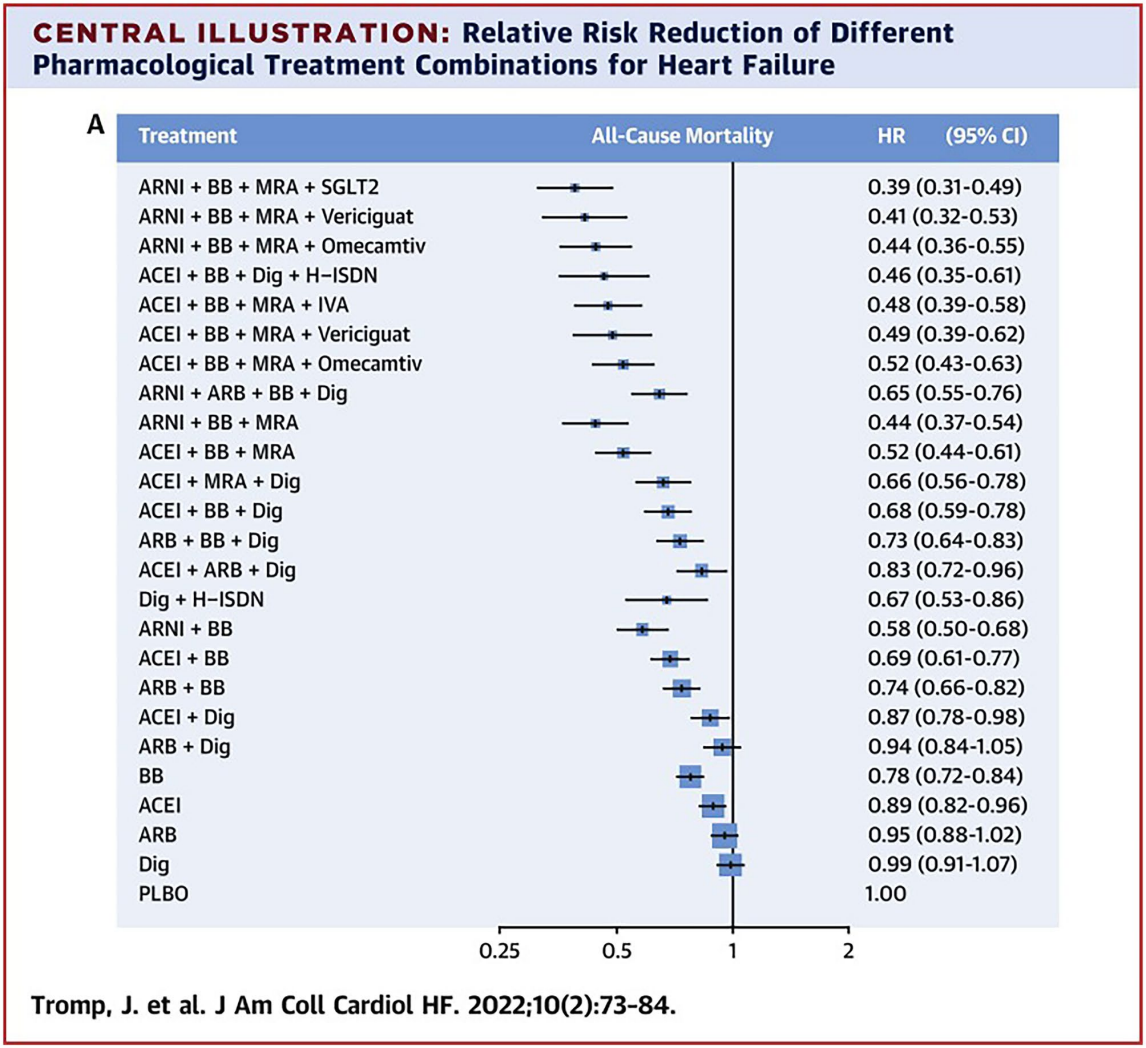
Relative risk reduction of different pharmacological treatment combinations for heart failure. Reprinted from JACC heart failure, volume 10, issue 2, Tromp et al., a systematic review and network meta-analysis of pharmacological treatment of heart failure with reduced ejection fraction, pages 73–84, copyright 2023, with permission from Elsevier. ACEi, angiotensin-converting enzyme inhibitor; ARB, angiotensin receptor blocker; ARNi, angiotensin receptor-neprilysin inhibitor; CV, cardiovascular; H-ISDN, hydralazine-isosorbide dinitrate; HF, heart failure; HFrEF, heart failure with reduced ejection fraction; HHF, hospitalization for heart failure; LVEF, left ventricular ejection fraction; MRA, mineralocorticoid receptor antagonist; NYHA, New York Heart Association; SGLT2i, sodium glucose cotransporter-2 inhibitors

To further illustrate the additive effect of GDMT, a 2020 analysis showed an additional 4.4 life years could be gained for 65-year-old patients receiving all four drug classes compared to just an ACEi/ARB and BB [35]. Furthermore, a large 2022 systematic review and network meta-analysis of the benefit of simultaneous treatment with all four drug classes compared to no drug treatment found a HR of 0.39 [95% CI 0.31–0.49], as seen in Fig. 2 [36]. Secondary analysis indicated that treatment with the four drug classes compared to no treatment resulted in 5 additional life-years for the 70-year-old patient [36]. These analyses further establish the significant and compounding life-saving effects that GDMT offers.

## Rapid drug sequencing and implementation strategies

In terms of implementation speed, evidence suggests that rapid drug initiation and fast up-titration is superior to a more gradual approach [37, 38]. Most RCT’s of the major drug classes demonstrated a rapid significant treatment effect, as early as 2–4 weeks after the initiation of therapy [20]. An analysis by Shen et al. used data from these large trials to model different sequencing strategies and found that faster compared to slower drug sequencing reduced the number of hospitalizations or CV death by as much as 47 per 1000 patients [37]. In order to confirm these results in a clinical trial, STRONG-HF randomized hospitalized patients with acute HF in 87 hospitals in 14 countries to either high-intensity care with a close follow-up regime, frequent natriuretic peptide measurements, and rapid up-titration within 2 weeks, or usual care [38]. In total, 542 patients were randomized to high-intensity care, while 536 were assigned to usual care. At 90 days, target dose was reached in 55% versus 2% of patient for RASi, 49% versus 4% for BB, and 84% versus 46% for MRA in the high-intensity care and control groups, respectively. Although serious adverse events were similar between the groups, the high-intensity treatment group had higher rates of adverse events at 90 days as compared to the control group at 41% and 29%, respectively. Due to a recommendation by the data safety monitoring board, the study was stopped early because of the large treatment effect of the high-intensity care intervention. At 180 days, the composite endpoint of mortality or hospitalization differed significantly between the groups at 15.2% for the high-intensity care group versus 23.3% for the control group. With regards to the implementation strategy, STRONG-HF initiated patients on all drug classes at medium strength doses during hospitalization with up-titration to target dose within 2 weeks after randomization [22, 39]. Despite the strengths of STRONG-HF, it should be recognized that a potential bias in the choice of treatment could have occurred since the trial had an open-label design. Also, the baseline level of GDMT was low and the very strict regimen is likely not suited for many health care systems. Moreover, one can question several centers that participated in the trial that had no heart failure clinic, or structured support (where tolerance can be a gamble), or access to ARNi/SGLT2. However, despite the amount of clinic visits being dissimilar between the groups, this did not lead to significantly more alterations in diuretic therapy.

GDMT has traditionally been implemented by starting the four drug classes in the consecutive order by which they were added to the guidelines, meaning starting ACEi first, then BB, followed by MRA, changing ACEi to ARNi, and lastly, initiating SGLT2i. Usually, a drug class is up-titrated to the maximally tolerated dose before moving on to the next. For example, the 2008 ESC guideline recommended up-titrating ACEi until the patient was clinically stable, then adding BB, and finally MRA in case of persisting symptoms [28]. The 2016 guideline instead recommends initiating ACEi and BB simultaneously, and adding MRA only if patients were still symptomatic [40]. Not only are there limited theoretical grounds for this traditional sequencing method, but a sequential step-by-step approach can take many months before optimal medical therapy is achieved. As suggested by STRONG-HF, rapid drug initiation and up-titration may save lives and reduce hospitalizations [38]. While the 2021 ESC HF guideline no longer offers clear recommendations in terms of sequencing strategies, novel rapid sequencing approaches have been proposed to shorten the time it takes to reach optimal medical therapy. For example, Packer and McMurray recommend a strategy involving simultaneously starting a BB and SGLT2i, rapidly followed by ARNi and MRA within 2 weeks [4, 20]. While BB offer a large benefit regarding the reduction of sudden cardiac death, the negative inotropic effect of BB can initially increase fluid retention and therefore worsening HF. The authors therefore propose the simultaneous initiation of SGLT2i to not only benefit from the treatment effect of two drug classes, but also because the diuretic effect of SGLT2i might improve the safety and tolerance of BB by mitigating fluid retention. Additionally, the use of ARNi and SLGT2i can reduce the risk of MRA induced hyperkalemia, possibly improving MRA adherence in the long term [41].

Alternatively, the JAMA viewpoint by Greene et al. suggests initiating all drug classes simultaneously at low doses, followed by up-titrating to target dose [22]. The authors argue that because de novo HF patients in the EPHESUS trial were generally able to tolerate simultaneous initiation of ACEi, BB, and MRA therapy after an acute myocardial infarction, there is no reason to assume tolerability would be significantly less for other HFrEF patients [42].

Finally, Rosano et al. identify specific patient subgroups based on their physiological profiles and recommend adjusting the sequencing strategy based on that respective profile [21]. Examples of such an individually tailored strategy would be initiating SGLT2i and MRA in patients with low blood pressure and a low heart rate, whereas patients with normal blood pressure and a high heart rate might be started on BB and ARNi. An overview of the different sequencing strategies is provided in the graphical abstract.

In order to gauge how sequencing of the four drug classes is done in real-world clinical practice, Fauvel et al. set out an international survey that was completed by 615 cardiologists from a variety of practice types [43]. Interestingly, despite the suggested, newer, approaches to drug sequencing, most respondents favored traditional sequencing over more novel implementation methods. However, it is important to state that 84% of the questioned cardiologists thought that starting all four drug classes during one initial hospitalization was realistic. Regardless, it should be recognized that even when traditional sequencing is the preferred drug implementation method, speed is of the essence. Whichever drug initiation and titration strategy is chosen, it is paramount that all four drug classes are initiated and up-titrated as close to target dose and as quickly as possible.

## Real-world adherence to guideline-directed medical therapy: a call to action for adequate implementation

The current GDMT for HFrEF is based on the maximum target doses used in the aforementioned RCTs. However, most of these trials have been conducted on predominantly younger, ethnically white male patients with less comorbidities compared to the average heart failure patient seen in clinic. This potentially causes a problem regarding generalizability and begs the question whether achieving target dose of all four drug classes is feasible for all patients [44]. Worldwide, multiple large-scale registries have reported on the relatively poor use of GDMT among HFrEF patients, all of which were conducted prior to the introduction of SGLT2i as part of standard GDMT [45–50]. Examining several large-scale registries from different countries provides real-world data on GDMT adherence and helps us achieve further insights into the potential barriers of GDMT initiation and up-titration. GDMT prescription rates and rates of achieved target dose are summarized in Table 3.

**Table 3:** GDMT prescription rates and rates of achieved target dose in registry studies

	**N**	**Mean age (years)**	**RASi**	**RASi td**	**BB**	**BB td**	**MRA**	**MRA td**
CHAMP-HF	2518	66.4	73.4%	16.8%	67.0%	27.5%	33.4%	76.6%
CHECK-HF	8360	72.3	84%	43.6%	86%	18.9%	56%	52.0%
ASIAN-HF	5276	59.6	77%	17%	79%	13%	58%	29%
ESC-LT (Spanish)	2834	65	92.6%	ACEi 16.2%/ARB 23.3%	93.3%	13.2%	74.5%	23.5%
QUALIFY	7092	63.1	86.7%	ACEi 27.9%/ARB 6.9%	86.7%	14.8%	69.3%	70.8%
Savarese 2021	68,172	65–75	-	ACEi 15%/ARB 10%/ARNI 30%	-	12%	-	-

*ACEi* angiotensin-converting enzyme inhibitor, *td* target dose, *ARB* angiotensin II receptor blocker, *ARNi* angiotensin receptor–neprilysin inhibitor, *BB* beta-blocker, *MRA* mineralocorticoid receptor antagonist

## CHAMP-HF

The 2018 CHAMP-HF registry was a prospective multi-center study of chronic HFrEF patients with LVEF < 40% in the USA [45]. Strikingly, only 1.1% of patients received triple therapy with RASi, beta-blocker and MRA, all at target dose. An absolute contra-indication for one of the drug classes was present in only < 2% of patients. Prescription rates differed significantly between specialties. For example, BB usage was 42.3% vs 70.5%, between family medicine/internal medicine and cardiology practices, respectively. Generally, old age, low blood pressure, high NYHA class and poor kidney function were patient factors associated with lower medication prescription rates and lower doses.

## CHECK-HF

The 2019 CHECK-HF registry was a large-scale multi-center cross-sectional study in The Netherlands [46]. Overall, only a third of the patients had triple GDMT therapy. However, the rate of triple therapy varied from 16 to 76% between centers, which could not be properly explained by differences in baseline characteristics. [51]. The rates of contra-indications for drug classes were also notably different between hospitals. BB contra-indication was, for example, recorded in 3.3% of patients, ranging from 0 to 27% between centers. The same was true for ACEi, which was recorded as being contra-indicated in 9.4% of patients, ranging from 0 to 36% between centers.

## ASIAN-HF

The 2018 ASIAN-HF registry was a prospective multi-national registry across 11 Asian countries [47]. Physiological barriers to uptritation alone did not explain the lack of GDMT adherence, because only 2% of all patients were hypotensive and only 6% had bradycardia. An eGFR < 30 was associated with decreased RASi and MRA prescription rates, whereas an eGFR < 15 resulted in lower BB use. Furthermore, a higher BMI was associated with higher rates of GDMT. Lastly, prescription rates also differed significantly between countries, with high-income countries being associated with improved GDMT adherence compared to low-income countries.

## ESC-LT

In the Spanish sub-cohort of the large-scale ESC LT registry, triple therapy was prescribed to 65.4% of patients with an LVEF < 35% [52]. With regards to ACEi, 4% of patients were intolerant or had a contra-indication, while 3.4% were undertreated. Undertreatment was identified in 1.8% of patients for BB, and 19.0% for MRA. For those who did not achieve target dose, about a third of patients were reported to still be in the titration phase. However, the study did not report on how long patients had been in this phase. Since all included patients had chronic HF, the percentage of patients still in titration phase should be significantly lower. It is therefore possible that patients reported to be in the titration phase could in reality be undertreated. Symptomatic hypotension was another frequent reason for not reaching target dose that was listed in 31.0%, 32.0%, and 19.6% for ACEi, ARB, and BB, respectively. For MRA, hyperkalemia was given as the reason for not achieving target dose in 10.4% of patients.

### Qualify global survey

This 2016 global prospective study was conducted at 547 centers in 36 countries [49]. Patients had chronic or worsening HF with an LVEF < 40%. For the patients not receiving an MRA or BB, it was scored as “not indicated” in 61.8% and 35.3% of cases, respectively. Geographic area resulted in different GDMT adherence rates. A secondary paper analyzing dose data at baseline and at 18 months follow-up found multiple patient factors to be associated with higher rates of up-titration, such as younger age, higher blood pressure, higher BMI, higher heart rate and less comorbidities [53].

### Savarese 2021

A multi-national observational cohort study conducted in Sweden, the UK, and USA looked at patients with a recent heart failure hospitalization who started at least one heart failure drug [50]. Across all three countries, up-titration rates were low and discontinuation rates were high. Discontinuation rates were 55% for ACEi, 33% for ARB, 24% for beta-blockers, and 27% for ARNi. Overall, age > 70 years and chronic kidney disease were associated with poorer GDMT adherence. However, also in patients < 70 years of age without chronic kidney disease target dose titration rates were insufficient.

### Barriers for GDMT implementation

#### Patient factors

It is often suggested that old age and frailty are the main causes of poor GDMT initiation and up-titration. This suggestion is supported by the registries, which generally showed poorer GDMT adherence in older patients with low blood pressure, poor kidney function and other comorbidities [45, 46, 50, 53]. Furthermore, in STRONG-HF patients were followed up and monitored closely while being up-titrated according to protocol [38]. Although patients in the high-intensity treatment group reached target dose significantly more as compared to the control group and patients in the registries, target dose was still not achieved in 100% of patients due to patient factors. Therefore, patient factors seem to be an important aspect of poor GDMT adherence. However, it has not been prospectively tested if it is justified to aim for lower target doses in such patients. In fact, the large RCTs hardly found interactions of the treatment effect with age or comorbidities. On another note, although not directly apparent from registry data, patient adherence to prescribed therapy is generally not optimal [54]. Multiple factors have been identified for this, such as a perceived lack of effect, mental health conditions, and poor health literacy [55]. The poor prognosis of HF justifies a high level of effort from both the patient and health care provider to achieve and maintain optimal medical therapy. The urgency of implementing life-saving therapy as soon as possible is already established in other fields such as oncology. For example, in many types of cancer with survival rates comparable to heart failure, side effects of therapy are more easily accepted by the patient and health care provider compared to HF medication that significantly improves survival and reduces hospitalization rates.

### Health care system factors

In CHECK-HF patients received GDMT more often compared to CHAMP-HF, which represent the Dutch and USA heart failure populations, respectively [45, 46]. This difference cannot be explained properly by patient characteristics, because the Dutch cohort was older and had worse kidney function compared to the US population. Possibly, the countries’ different health care systems are an influencing factor. In the Netherlands health care is partly paid for by the government and it is mandatory for every citizen to have health insurance, while in the USA, not all patients have access to health insurance. Therefore, high healthcare costs could be a barrier for patients to add more medication or to switch to more expensive medication [55]. Additionally, both ASIAN-HF and the QUALIFY global survey found geographical area to be associated with GDMT adherence [47, 49]. Although the reasons for these inter-country differences are not clear, they may be speculated to be caused by differences in healthcare provider education and views, insurance and government policies, limitations regarding healthcare access, or differences in the general health care system. Although STRONG-HF demonstrated the effectiveness of high-intensity care and rapid up-titration, it must be acknowledged that real-world implementation of such high-intensity care might not be feasible for all countries or health care systems.

### Local hospital/clinician factors

CHECK-HF found significantly different GDMT prescription rates between hospitals that were not attributable to patient characteristics [51]. All patients were included in Dutch hospitals, in a country where expensive medication does not increase costs for patients. This suggests that local hospital protocols and differences in care setting could be important factors in how patients are treated [55], even though all healthcare providers should follow the ESC HF guideline.

Besides hospital factors, the individual clinician very likely plays a key role in the quality of provided care and GDMT prescription rates. First of all, there is the risk-treatment paradox. This means that the sickest and most frail patients receive less intensive treatment, although they may profit most from GDMT in absolute terms [56]. In an analysis from GWTG-HF, at-risk patients received less evidence-based therapy compared to lower-risk patients, even when corrected for contraindications [57]. This phenomenon could be explained not only by gaps in evidence, but also by the clinicians’ concern for drug-related adverse events outweighing the benefits for the patient [56]. Secondly, in the ADDress your Heart survey, cardiologists were presented fictitious cases to examine their adherence to the guideline [58]. Although guideline awareness was high, only < 25% recommended GDMT. Guideline complexity was considered to be an important barrier for correct guideline implementation according to the respondents [58]. Moreover, an integrative review on the factors of clinical inertia states that the health care provider’s disagreement with the guidelines limits guideline adherence, as well as the perceived lack of applicability of the guidelines to the individual patient [59].

Clinical inertia has been written about extensively and is generally defined as a lack of treatment intensification in a patient not at evidence-based goals for care [60]. As an example, there is a mismatch between the high sense of urgency HF warrants and the percentage of chronic HF patients still in the titration phase as described by the ESC-LT registry. Furthermore, secondary analysis of the GUIDE-IT trial showed that only 15.5% of patients received at least 50% of triple therapy target dose, even with the use of NT-proBNP guidance. In 11.1% of the clinic visits the investigators reported that therapy was not started or up-titrated because the patient was already “clinically stable” [61]. If patients are clinically stable or little symptomatic and satisfied with their current treatment, it can occur that current medication is maintained instead of further up-titrating or initiating new drug classes. Besides patient preference, this can be due to multiple factors relating to the clinician. These clinician factors include a lack of time, clinician burnout and apathy, disproportionate fear of risks, formulary archaism, and perceived lack of guideline applicability to the individual patient [62]. However, it must be acknowledged that it remains difficult to distinguish true clinical inertia from adequate individually tailored care.

## Future insights

**Fig. 3:**
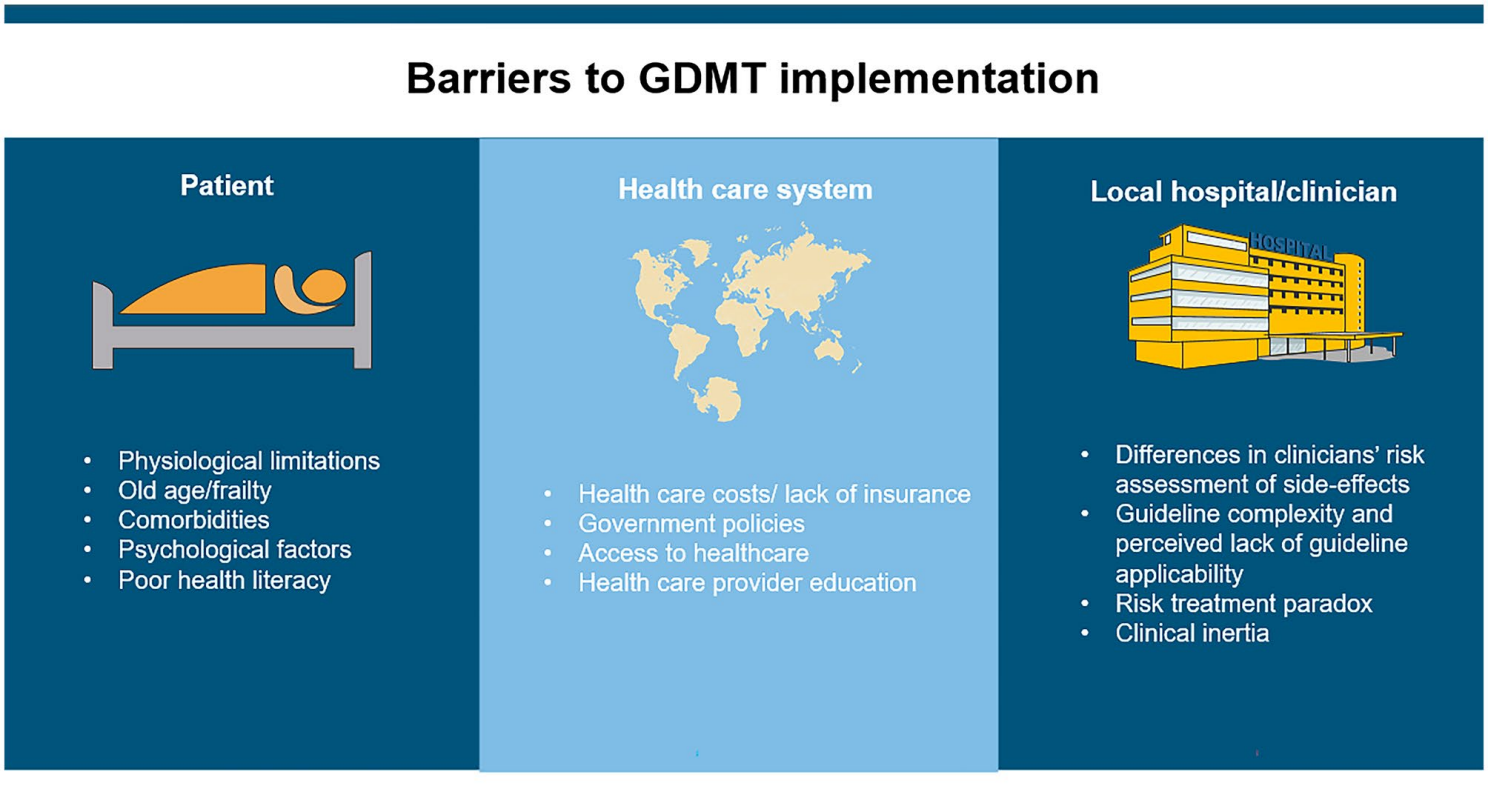
Barriers to GDMT implementation. The figure was partly generated using Servier Medical Art, provided by Servier, licensed under a Creative Commons Attribution 3.0 unported license. GDMT, guideline-directed medical therapy

Despite the high mortality of HFrEF, GDMT adherence was low across multiple countries, healthcare systems, patient subgroups, and healthcare providers, as summarized in Fig. 3. Although large registries often report on lacking rates of GDMT, specifics around the question “why?” are lacking. Over the years, HFrEF treatment has expanded significantly in terms of drug classes, but now the focus should shift towards rapid and complete implementation of all four live-saving drug classes.

### Quicker up-titration of life saving drugs: rapid sequencing strategies

First, strategies for improving GDMT adherence should be sought. Conscious and scheduled up-titration regiments as described in the STRONG-HF trial, or smarter sequencing strategies as described by Packer and McMurray, can improve GDMT adherence [20, 38]. Simultaneous initiation of drugs as recommended by Greene et al. or adjusted sequencing strategies based on patient characteristics as described by Rosano et al. could improve the speed and efficacy of GDMT implementation [21, 22]. However, the implications of these strategies have yet to be tested in both scientific studies and real-world clinical practice.

It is presently still unknown if clinicians must strive to titrate all patients to the same target dose or if certain subgroups benefit from adjusted and possibly lower target doses. Since patients in the large RCT’s were younger and had less comorbidities compared to the average real-world patient, it could be hypothesized that for older and more frail patients the optimal therapeutic benefit might be achieved at lower doses. Indirect evidence from a meta-analysis on individual data of NT-proBNP guided trials suggests that more intensified therapy might not result in better outcomes in patients with significant co-morbidities [63].

### Definition of true intolerability

As highlighted in this review, the interpretation of intolerability varies significantly between healthcare professionals. To a large extent, this is related to the fact that true intolerability has not yet been tested. Contraindications for certain drugs are often defined based on the exclusion criteria applied in RCTs but not based on scientific evidence. For example, the use of ARNi is discouraged in patients with eGFR < 30 simply because such patients have been excluded from the according trial [4, 16]. Similarly, SGLT2i may not be used with eGFR < 20–30 although they have proven reno-protective effects [1, 2, 4]. To overcome this uncertainty, appropriate trials are required to test if such assumed intolerability or contraindication is scientifically sound. In addition, registries including patients that receive treatment despite some (relative) contraindications may help to address this question.

### The urgency for better GDMT implementation: the need for all four

With the introduction of a fourth drug class as standard therapy for all HFrEF patients, in addition to other drugs such as diuretics, antiarrhythmic therapy, ivabradine, digoxin, and more recently verigicuat, the use of best therapy has become increasingly complex [4].

Therefore, future research should focus on achieving a better understanding of the barriers of drug initiation, use, and up-titration. For example, the retrospective chart analysis CHART-HF will analyze patients with a worsening heart failure event and analyze the clinician-reported reasons for GDMT non-adherence [64]. Additionally, the TITRATE-HF registry aims to prospectively record every step of the titration process, and register the barriers of up-titration in heart failure patients in The Netherlands. Both studies will not only register reasons for medication changes, but also detail the reasons for not initiating or up-titrating medication. New registries will simultaneously provide insight into the real-world implementation of the latest drug class, SGLT2i, since this data is currently limited.

### Infrastructure needed for GDMT implementation

Finally, an important cornerstone of HF care is the infrastructure needed to facilitate healthcare providers in helping their patients. An important threat of the infrastructure is the collective burden on healthcare providers, due to the increasing workload and scarcity of healthcare personnel. This burden is leading to high rates of burnout and apathy in healthcare providers, which makes providing proper HF care on its own already challenging [62, 65, 66]. Providing proper HF care includes having an outpatient clinic with specialist HF nurses that educate and counsel the patients, perform checks and follow-up visits, and collaborate with physicians from both inside and outside the hospital.

To decide what is needed to improve implementation of GDMT in terms of infrastructure, one should look at the lessons learned from STRONG-HF [38]. The high intensity care group had a very strict, protocolized, HF care regime with additional visits to monitor patients, which may have played an important role in addition to the effect of rapid up titration [67]. While the burden of healthcare personnel is generally speaking already high, it seems that frequent visits or at least contacts with the patient play a major role in starting and up-titrating GDMT.

Other, more modern, infrastructure-based initiatives to improve GDMT implementation include electronic health record (EHR) based tools to encourage clinicians to start and up-titrate GDMT through encouraging prompts and visualizations embedded within the EHR [68, 69], and integrating risk-prediction tools in established care pathways [70]. These modern approaches can help reduce the burden on healthcare personnel by providing clinicians clear choices and suggestions on how to proceed with the patient in front them.

The main message that can be taken from these studies is that frequent visits and contacts with the patient are important, especially during the first few weeks after starting or up-titrating a drug class. However, more effective and modern approaches are urgently needed to reduce workload and make the infrastructure of HF care sustainable for the future.

## Conclusion

Although medical therapy for heart failure will always remain an individually tailored art, future research may offer clinicians additional much needed tools to help further optimize their craft.

## Data Availability

No new data were generated or analyzed in support of this research.
